# Model selection with multiple regression on distance matrices leads to incorrect inferences

**DOI:** 10.1371/journal.pone.0175194

**Published:** 2017-04-13

**Authors:** Ryan P. Franckowiak, Michael Panasci, Karl J. Jarvis, Ian S. Acuña-Rodriguez, Erin L. Landguth, Marie-Josée Fortin, Helene H. Wagner

**Affiliations:** 1 Environmental & Life Sciences Graduate Program, Trent University, Peterborough, Ontario, Canada; 2 Department of Natural Resources Management, Texas Tech University, Lubbock, Texas, United States of America; 3 Department of Biology, Southern Utah University, Cedar City, Utah, United States of America; 4 Centro de Ecología Molecular y Aplicaciones Evolutivas en Agroecosistemas (CEM), Instituto de Ciencias Biológicas, Universidad de Talca, Talca, Chile; 5 Departamento de Biología, Facultad de Ciencias, Universidad de La Serena, La Serena, Chile; 6 Division of Biological Sciences, University of Montana, Missoula, Montana, United States of America; 7 Department of Ecology & Evolutionary Biology, University of Toronto, Toronto, Ontario, Canada; Universita degli Studi di Trento, ITALY

## Abstract

In landscape genetics, model selection procedures based on Information Theoretic and Bayesian principles have been used with multiple regression on distance matrices (MRM) to test the relationship between multiple vectors of pairwise genetic, geographic, and environmental distance. Using Monte Carlo simulations, we examined the ability of model selection criteria based on Akaike’s information criterion (AIC), its small-sample correction (AICc), and the Bayesian information criterion (BIC) to reliably rank candidate models when applied with MRM while varying the sample size. The results showed a serious problem: all three criteria exhibit a systematic bias toward selecting unnecessarily complex models containing spurious random variables and erroneously suggest a high level of support for the incorrectly ranked best model. These problems effectively increased with increasing sample size. The failure of AIC, AICc, and BIC was likely driven by the inflated sample size and different sum-of-squares partitioned by MRM, and the resulting effect on delta values. Based on these findings, we strongly discourage the continued application of AIC, AICc, and BIC for model selection with MRM.

## Introduction

A primary goal of landscape genetics is to determine the relative influence of landscape composition (e.g., amount of habitat), configuration (spatial arrangement of habitat patches), and matrix quality (landscape between habitat patches) on patterns of gene flow, genetic discontinuities and population genetic structure [1–5]. Gene flow may be restricted by geographic distance (isolation-by-distance) and by resistance of land-cover types to movement (isolation-by-resistance). Because gene flow depends on what lies between patches and not the conditions within patches (sampling locations), hypotheses are expressed in terms of pairwise distances between patches [6]. While the genetic data are collected within patches, genetic differentiation resulting from restricted gene flow is quantified in terms of pairwise genetic distances. Hypotheses concerning the association of pairwise distances between sampling units (i.e., genetic, geographic, environmental, or temporal distances) are often analyzed using Mantel tests [7] or its derivatives, such as partial Mantel test [8] and multiple regression with distance matrices (MRM) ([9–11], for examples see [12–14]). Competing hypotheses are typically defined in either of two ways: (1) each hypothesis is represented by a single distance matrix D_x_ that integrates hypothesized effects of multiple landscape features, or (2) each factor p is represented by its own distance matrix D_p_ and each hypothesis is defined by a set of predictor matrices [6,10,15]. Various model selection approaches have been proposed for identifying the model that best explains the observed spatial genetic structure and assessing the level of support for each competing hypothesis [8,16–23], but the accuracy and reliability of these approaches remain a topic of considerable debate in the context of spatial analysis (e.g., [24,25]).

Model selection procedures based on Akaike’s information criterion (AIC) [26], its small sample size correction (AICc) [27], and the Bayesian information criterion (BIC) [28] have been suggested as a potential alternative to traditional statistical hypothesis testing for analyzing landscape genetic data [5,29], and these methods have been used increasingly with the Mantel test [30–33] and MRM [23,34–41]. AIC and AICc are information theoretic indices and aim to identify the fitted model with the minimum loss of Kullback-Leibler (K-L) information compared to the full reality, whereas BIC aims to identify the model with the fewest parameters that is nearest to the truth as measured by K-L distance [17,18]. In practice, AIC has a tendency to include too many predictors (overfitting) irrespective of sample size, whereas BIC has a tendency towards underfitting that increases with sample size [42]. AIC, AICc, and BIC values are not directly interpretable due to unknown scaling constants and strong dependence on sample size, but instead rely on delta Δ_*i*_ values, which represent the difference in AIC, AICc, or BIC values between candidate model *i* and the selected best model (i.e., Δ_*i*_ = *AIC*_*i*_ − *AIC*_*min*_), and provide a quantitative measure of support for each competing hypothesis [17,18]. In situations where more than one model from the candidate set of models is supported by the data, model averaging procedures may be used based on model weights $w_{i}=\text{exp}\left(-1/2\;\Delta_{i}\right)/\Sigma_{r=1}^{R}\text{exp}\left(-1/2\;\Delta_{r}\right)$ ([17], p. 75). Because each model *i* is weighted with respect to all other models *r* in the entire set of candidate models *R*, model averaging generally results in more robust parameter estimates and model predictions [17,18].

A linear relationship *r*_xy_ between two normally distributed variables *x* and *y* observed at *n* sampling locations translates into a linear relationship between two vectors of pairwise distances D_x_ and D_y_, where each element in D_x_ is the difference (*x*_j_−*x*_i_) between two values of *x* observed at locations *i* and *j*, with a linear (Mantel) correlation between D_x_ and D_y_ slightly smaller than *r*^2^_xy_ [43]. Here, we refer to the analysis of the relationship between *x* and *y* as *node-based* analysis, and the analysis of the relationship between D_x_ and D_y_ as *distance-based* analysis [6]. Mantel tests evaluate the (full or partial) correlation between D_x_ and D_y_, whereas MRM performs regression analysis of D_y_ on one or more predictors D_x_. While distance-based analysis is a round-about and inefficient way for assessing the linear relationship between *x* and *y* where node-based analysis can be applied, it is useful in cases where the predictor variable exists only in the form of pairwise differences [44]. In the case of hypotheses about landscape resistance to gene flow, the ecological distance between two sampling locations (predictor variable D_x_) depends on the resistance values of all land-cover types between the two locations, not on the values at the sampling locations.

MRM as a distance-based analysis differs from standard, node-based regression analysis in important ways [45], as it tests the relationship between two or more vectors of *N* = *n*(*n* − 1)/2 unique distance values derived from *n* independent observations. Thus, values are not independent, as each of the *n* original observations will contribute to *n* − 1 of the *N* values in the distance vector. This leads to several complications. (1) Due to the non-independence of pairwise observations, statistical significance tests must be based on appropriate permutation tests rather than parametric procedures (e.g., [43])–this is now routinely implemented. (2) Spatial autocorrelation may further jeopardize statistical significance testing by inflating type I error rates [24,25]. (3) MRM minimizes a different residual sum-of-squares (RSS) than linear regression of the node-based data from which the pairwise distances were derived [45]. Hence, even if based on the same original data, we should not expect to find the same parameter estimates. Indeed, the Mantel correlation *r*_*M*_, calculated from the *N* pairwise distances, is generally much lower than the corresponding Pearson correlation *r* calculated from the *n* original values [44,45]. (4) The non-independence of pairwise observations invalidates the use of AIC and similar measures in MRM [22]. This problem cannot be easily fixed by adjusting for inflated sample size, as the true degrees of freedom in distance matrices are unknown [22,46]. Hence, adjusting the sample size in the calculation of AIC, AICc, and BIC is not a recommended strategy. While these issues are known to exist, there is a lack of research that would allow authors and reviewers to judge the severity of the consequences of using AIC, AICc or BIC with MRM to assess the empirical support for competing models.

In this study, we used a simple Monte Carlo simulation approach to evaluate the behavior and performance of AIC, AICc, and BIC when applied with MRM. Rather than mimicking the full complexity, e.g., of landscape genetic data, we present an artificially ideal situation, where pairwise distances are derived from node-based data simulated as multivariate normal variables with known linear correlation structure and without complicating factors, such as spatial autocorrelation or collinearity, among predictor variables. This approach allowed us to use the results from node-based analysis as a benchmark for the results from distance-based analysis. We determined the ability of AIC, AICc, and BIC to (1) identify and provide empirical support (i.e., delta values Δ_*i*_ and model weights *w*_*i*_) for the correct, single-predictor model when confronted with a candidate set of models containing an increasing number of spurious predictors, and (2) identify the correct model with multiple predictors varying in strength of correlation (i.e., tapering effects) with the response variable. This study aims to address, in part, a current research priority in landscape genetics, which is to (1) evaluate how well various analytical approaches perform at identifying the relevant factors controlling gene flow in complex landscapes, (2) determine under what conditions they perform reliably, and (3) assess how they are affected by common violations of assumptions.

## Methods

Using a Monte Carlo simulation approach, we evaluate the ability of AIC, AICc, and BIC to identify the correct model when applied to MRM on distance transformed data. Simulations allowed us to compare multiple linear regression and MRM analysis under conditions where the relationship between the response and predictor variables in each simulated dataset were known. Simulated data sets consisted of six random variables sampled from a multivariate normal distribution with zero means and a pre-specified covariance matrix, where all diagonal values (variances) were set to one and all off-diagonal terms (covariances, i.e., expected correlations *ρ*_*ij*_) were set to zero unless specified otherwise below. First, node-based data were generated using the *mvrnorm* function in the MASS package [47] in R [48]. Each data set included a single response variable *y* and five predictor variables *x*_1_ − *x*_5_, with all predictors being independent of each other (i.e., no collinearity) and without spatial autocorrelation. These conditions present an ideal situation, where the model selection methods we wanted to test would be most likely to perform well. In a second step, node-based data were transformed into distance-based data. Data were simulated under two scenarios: (1) with a single meaningful predictor and four spurious predictors, and (2) with three meaningful predictors with decreasing effects on *y* and two spurious predictors.

*Node-based analysis*–In the first series of simulations, we examined the ability of AIC, AICc, and BIC to correctly penalize for spurious predictors, i.e., to select the correct model containing a single meaningful predictor variable and zero independent random variables, *y*_*i*_ = *β*_0_ + *β*_1_*x*_1_ + *ε*_*i*_, when confronted with a candidate set of models containing an increasing number of independent random variables. In each simulation run, the single meaningful predictor *x*_1_ was assigned an expected correlation of *ρ*_*xy*_ = 0.60 with the response *y*, whereas variables *x*_2_ − *x*_5_ were simulated to be independent of *y* (*ρ*_*xy*_ = 0.0). Following Peres-Neto et al. [49], we generated four incorrect models by sequentially adding four additional independent random variables *x*_2_ − *x*_5_ to the correct model with the meaningful predictor *x*_1_.

In the second series of simulations, we examined the ability of AIC, AICc, and BIC to identify the correct model across different levels of strength of correlation with the response variable. Specifically, we assessed the performance at correctly ranking models containing weak tapering effects incorporated in the data; keeping in line with the variable selection problem which is often the focus of multiple regression analysis [17,18]. We modified the correlation matrix used to generate the simulated data sets assigning expected correlation values of *ρ*_*xy*_ = 0.30, 0.25, 0.20 to variables *x*_1_ − *x*_3_, with *x*_1_ having the highest and *x*_3_ the lowest correlation with *y*. We simulated the remaining two variables *x*_4_ and *x*_5_ to be independent of *y* (*ρ*_*xy*_ = 0.0). The candidate set of models contained again five models, but in this series of simulations the correct model contained three meaningful predictors and zero independent random variables, *y*_*i*_ = *β*_0_ + *β*_1_*x*_1_ + *β*_2_*x*_2_ + *β*_3_*x*_3_ + *ε*_*i*_, where the linear effects of variables *x*_1_ − *x*_3_ on *y* were assumed to be additive. We performed regression analyses for both node-based simulations using the *lm* function in R [48]. We calculated AIC and BIC using the basic R functions *AIC* and *BIC*, and we calculated AICc using function *AICc* of R package ‘MuMIn’ [50].

*Distance-based analysis*–The six normally distributed random variables within each node-based data set (described above) were transformed into Euclidean distance matrices using the *dist* function in the R package ‘stats’, from which we extracted the lower-triangle values as a vector of *N* = *n*(*n* − 1) pairwise distances, which we subsequently analyzed with MRM. For pairs of linearly correlated normally distributed variables, we expected the correlation between distance-transformed variables to be less than the square of the node-based values [44]. The transformation into distance values can also introduce non-linearity of the relationship that further reduces linear correlation [45]. To account for the reduction in the strength of correlation caused by the distance transformation, we modified the correlation matrix to generate a second set of node-based data with higher expected correlation values of *ρ*_*xy*_ = 0.8 instead of 0.6, and {0.58, 0.52, 0.47} instead of {0.30, 0.25, 0.20}. These data were again transformed into a second set of Euclidean distance matrices (in this case, only the distance-based data were retained for analysis). Thus, we were able to evaluate the reliability of AIC, AICc, and BIC when applied with MRM on distance data with a reduced correlation derived directly from the raw data (low correlation set of distance vectors) and independently simulated distance data with an empirical correlation equal to the original raw data (high correlation set of distance vectors). The calculations for fitting an MRM model are no different than those for multiple regression with raw data, and thus we fitted the same five models used in the node-based analysis using the *lm* function in R [48], using the same functions to calculate AIC, AICc and BIC.

For each set of simulations, we determined the reliability with which model selection algorithms based on AIC, AICc, and BIC were able to identify the correct model when applied with MRM by the proportion of 1000 replicate data sets where we identified the correct model as the best model. To further understand how the number of observations *n* in the original raw data influences the behavior of AIC, AICc, and BIC, we ran each simulation with three different initial sample sizes, *n* = 30, 100, and 300. R code for generating and analyzing simulated data sets is available as S1 File.

## Results

### Simulations with an increasing number of spurious predictors

The first set of simulations resulted in empirical correlations between *y* and *x*_1_ for the node-based data that varied around the expected correlation of *ρ*_*xy*_ = 0.60, with mean = 0.598 (*sd* = 0.063) based on 1000 replicate simulations with a sample size of *n* = 100. Transformation to distance vectors reduced the average correlation to 0.322 and increased the standard deviation of empirical correlation coefficients to 0.078. Increasing the pre-specified covariance for the second set of distance vectors resulted in a mean correlation of distance vectors of 0.604 (*sd* = 0.062), closely matching the properties of the node-based correlations.

**Fig 1:**
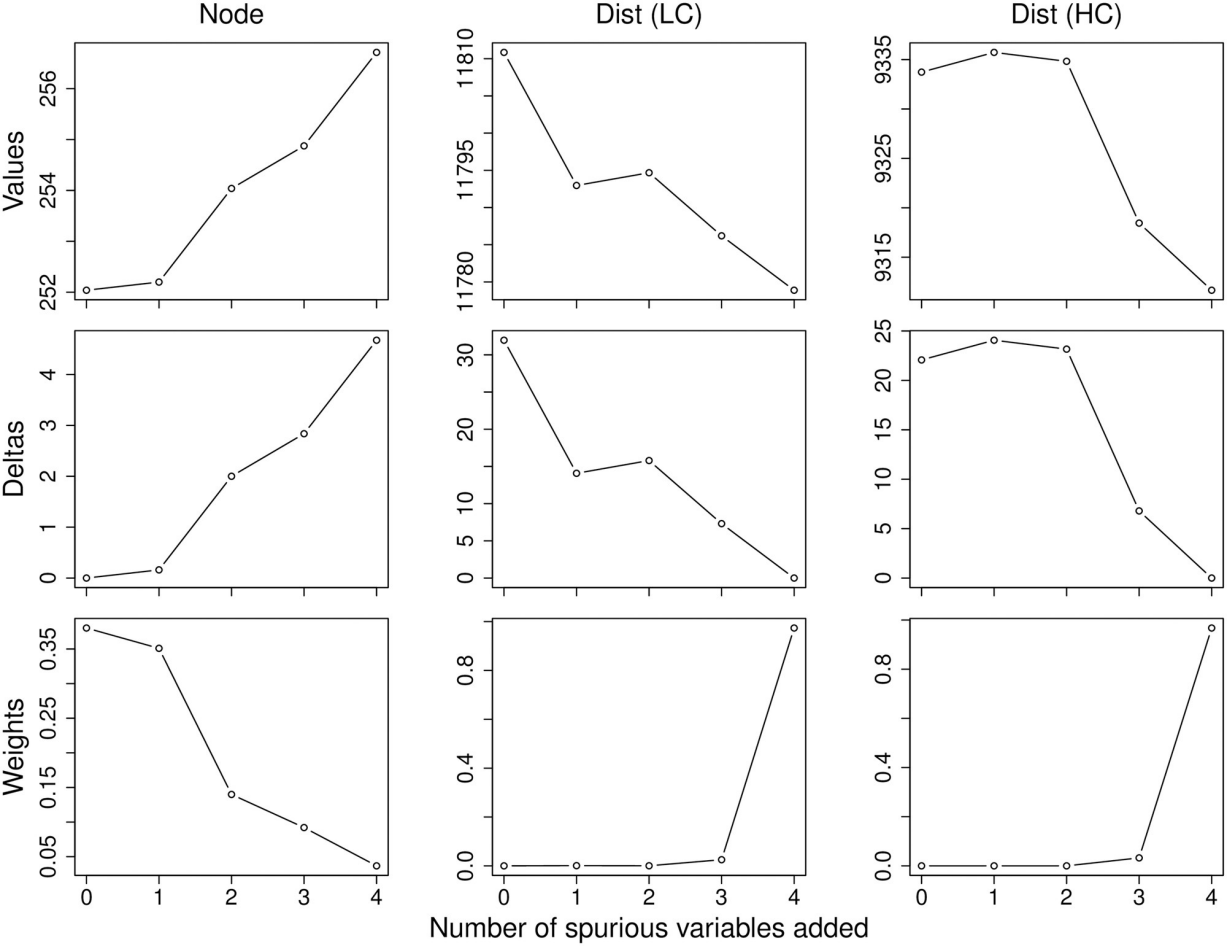
Results from a single simulation run. The absolute values (top row), delta values Δ_*i*_ (middle row), and model weights *w*_*i*_ (bottom row) for node-based analysis (Node: left column), distance-based analysis with low correlation (Dist (LC): middle column), and distance-based analysis with high correlation (Dist: (HC): right column) as a function of the number of spurious random variables added sequentially to the correct model with a single meaningful predictor *x*_1_ (*ρ*_*xy*_ = 0.6) for node-based, based on *n* = 100.

The results from a typical single simulation run with *n* = 100 (Fig 1) illustrate how the behavior of AIC changed markedly when used with MRM on distance transformed data (patterns for AICc and BIC were similar, not shown). For the node-based analysis (left column), the correct model with a single meaningful predictor and zero independent random variables, *y*_*i*_ = *β*_0_ + *β*_1_*x*_1_ + *ε*_*i*_, had the lowest absolute value (top row), and thus the lowest delta Δ_*i*_ value (middle row), with values for both increasing monotonically with each additional variable added to the model. The correct model also had the highest model weight *w*_*i*_ (bottom row).

When applied to MRM, the absolute values, and more importantly, the delta values Δ_*i*_, were considerably larger than those observed for the node-based regression analysis, with similar patterns for both sets of distance vectors (center column: low correlation, right column: high correlation). As in this example, the correct model often had the largest absolute value and, contrary to expectations, the values generally decreased with each additional variable added to the model. Moreover, delta values Δ_*i*_ not only reversed the rank order of the five candidate models, but also increased more rapidly between successive models than those reported for the node-based analysis, and thus, provided high weight *w*_*i*_ of support (bottom row) for the incorrect model, with the remaining models, including the correct model, receiving little support.

**Fig 2:**
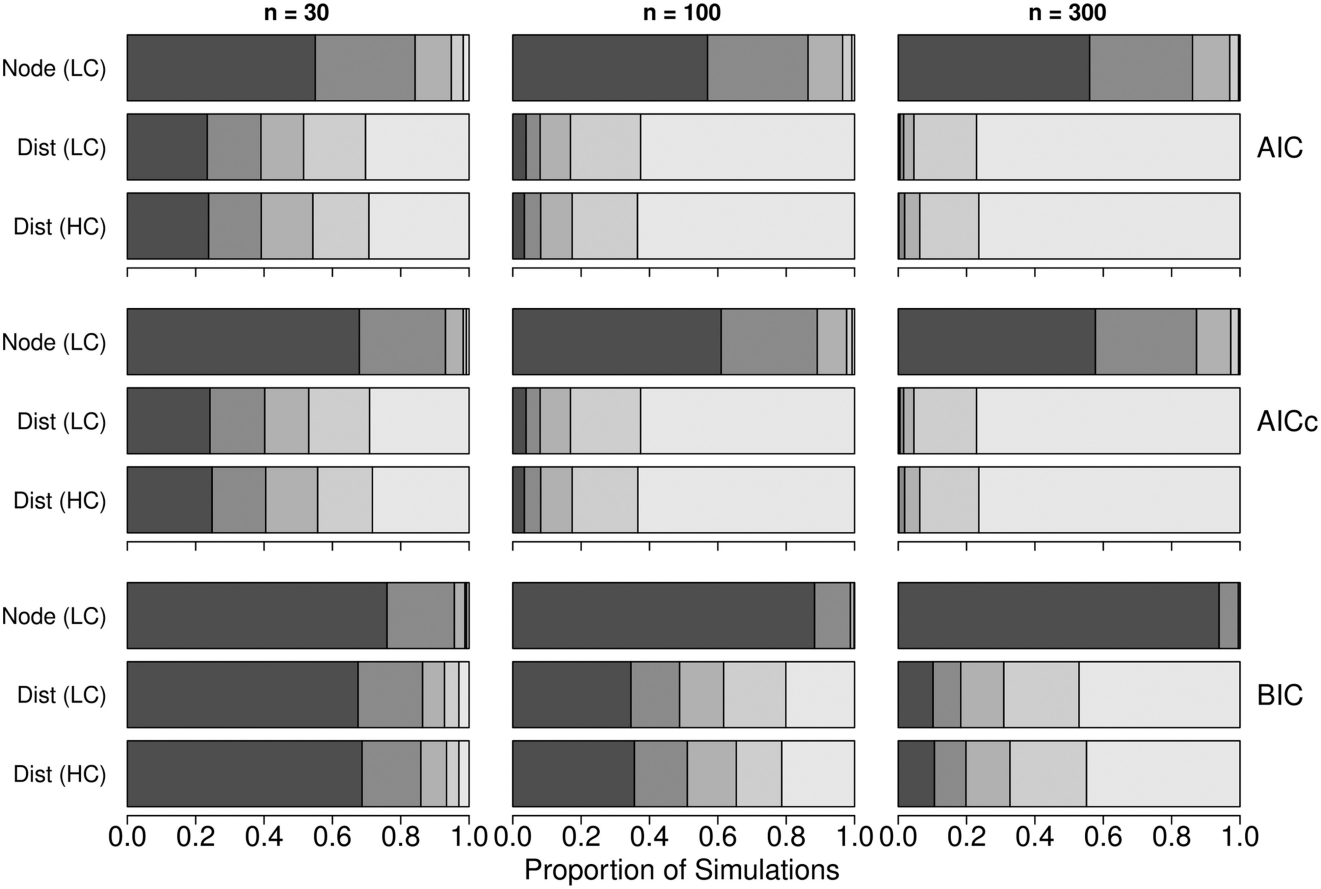
Proportional selection of the correct model by means of MRM among 1000 simulated data sets with a different number of spurious predictors. The proportion of 1000 simulated data sets where each of the five candidate models was selected as the best model using AIC (top row), AICc (middle row), and BIC (bottom row) with three different sample sizes of *n* = 30 (left column), *n* = 100 (middle column), *n* = 300 (right column) for the node-based analysis with low correlation (Node LC), the distance-based analysis with low correlation (Dist LC), and the distance-based analysis with high correlation (Dist HC). The correct model included only the single meaningful predictor *x*_1_ (black), whereas, the four additional models contained the single meaningful predictor *x*_1_ and one (dark grey), two (medium dark grey), three (medium light grey), and four (light grey) spurious variables (*x*_2_ − *x*_5_).

Across 1000 replicate simulations with an increasing number of spurious variables (Fig 2), AIC, AICc, and BIC applied with multiple regression on the original raw data (node-based analysis) were able to select the correct model as the best model in the majority of simulations, and the ability of all three criteria to identify the correct model generally increased with larger sample size *n*. Under this scenario, BIC performed more reliably than either AIC or AICc, selecting the correct model in more than 76 percent of simulations, whereas, AIC and AICc selected the correct model in less than 57 and 68 percent of simulations, respectively. These results serve as a benchmark for distance-based analysis.

When applied to distance-based analysis MRM, AIC, AICc, and BIC exhibited a strong bias toward selecting models containing spurious effects and, more surprisingly, the severity of this bias increased markedly with larger sample size *n*. For simulations run with *n* = 300, AIC and AICc selected the model with all four additional spurious variables in more than 76 percent of simulations (for both low and high correlation), whereas BIC selected this same model in 45 and 47 percent of simulations for distance vector data generated with low and high correlation, respectively.

### Simulations with multiple meaningful predictor variables

The second set of simulations resulted in empirical correlations between *y* and *x*_1_ − *x*_3_ for the node-based data that varied around the expected correlation of *ρ*_*xy*_ = 0.30, 0.25, and 0.20, with mean = 0.299, 0.247, and 0.198 (*sd* = 0.092, 0.094 and 0.096) based on 1000 replicate simulations with a sample size of *n* = 100. Transformation to distance vectors reduced the mean correlation to 0.078, 0.053, and 0.037 (*sd* = 0.063, 0.060, and 0.055) for the low-correlation data set (LC). Increasing the pre-specified covariance for the second set of distance vectors (HC) resulted in a mean correlation of distance vectors of 0.30, 0.24, and 0.19 (*sd* = 0.080, 0.076, and 0.077), closely matching the properties of the node-based correlations.

**Fig 3:**
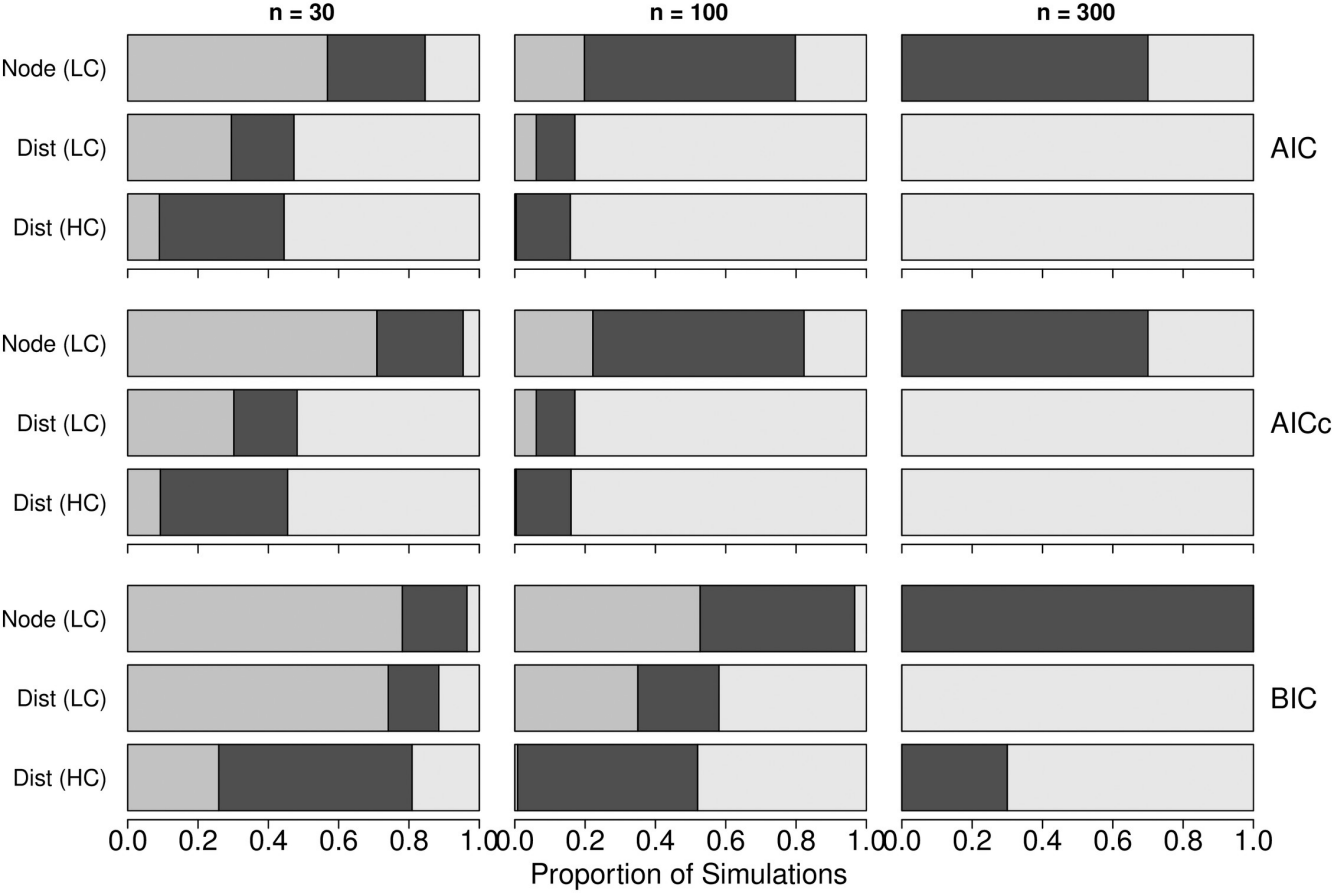
Proportional selection of the correct model by means of MRM among 1000 simulated data sets for different levels of correlated predictors. The proportion of 1000 simulated data sets where each of the five candidate models were selected as the best model using AIC (top row), AICc (middle row), and BIC (bottom row) with three different sample sizes *n* = 30 (left column), *n* = 100 (middle column), *n* = 300 (right column) for the node-based analysis with low correlation (Node LC), the distance-based analysis with low correlation (Dist LC), and the distance-based analysis with high correlation (Dist HC). We were primarily interested in determining whether AIC, AICc, and BIC selected the correct model containing three meaningful variables with tapering effects (black) or selected an underfitted (dark grey) or overfitted (light grey) model.

For the 1000 replicate simulations run with three meaningful predictors with tapering effects (Fig 3), AIC, AICc, and BIC applied with multiple regression on the original raw data (node-based analysis) exhibited considerable uncertainty in selecting the correct model, particularly for small sample size *n* = 30, though the ability of all three criteria to identify the correct model improved with larger sample size *n*. In simulations run with *n* = 300, AIC and AICc performed less reliably than BIC; both measures identifying the correct model in 70 percent of simulation, whereas, BIC identified the correct model in 100 percent of simulations. Again, these results of node-based analysis provide a benchmark for the results from distance-based analysis.

When applied to distance-based analysis with MRM, AIC, AICc, and BIC were unable to reliably identify the correct model regardless of sample size *n* or strength of correlation used to generate the raw data. The overall performance of AIC, AICc, and BIC in distance-based analysis decreased markedly with larger sample size *n*. AIC and AICc exhibited a strong bias toward selecting the model containing all five variables (i.e. full model), but the bias was slightly less severe for BIC data generated with high correlation.

## Discussion

When applied with MRM (distance-based analysis), model selection procedures based on AIC, AICc, and BIC were unable to reliably rank candidate models or consistently identify the correct model, and thus often led to incorrect inferences about the relationships between response and predictor variables. All three criteria exhibited a systematic bias toward selecting unnecessarily complex models and indeed would often select the full model (with two or four spurious predictors included depending on the scenario) as the best model. The absolute and relative values (i.e., delta values Δ_*i*_) of AIC, AICc, and BIC exhibited very different behavior that interfered with the ability of each criterion to correctly rank candidate models. The large delta values suggested a high level of support for the incorrectly selected best model, and thus provided little support for other models in the candidate set, including the correct model. The observed change in behavior goes beyond the general bias toward overfitting often cited for AIC (and AICc) when comparing models containing a small number of predictors with large effect [17,18]; a pattern that can be seen in the results from the node-based analysis. The observed bias became more pronounced when we increased the sample size *n* used to generate the original raw data, which is directly related to using *N* = *n*(*n* − 1)/2 pairwise distance values to calculate AIC, AICc, and BIC values for MRM on distance matrices. BIC performed slightly better, as its penalty increases with sample size, but this was not sufficient to correct for the problem in distance-based analysis. Manually adjusting for sample size in the calculation of AIC, AICc, and BIC may confer some improvement (S2 File; S1–S3 Figs) but cannot be recommended as it does not adequately address the problem of unknown degrees of freedom [46]. Thus, our results suggest considerable caution should be taken when interpreting and evaluating studies that have relied on model selection with MRM to assess the relationship between landscape features and patterns of gene flow [23,34–41].

Another substantial barrier to the application of AIC, AICc, and BIC with MRM is the non-independence of pairwise distances; this violates a basic assumption of linear regression analysis [51] and can strongly bias model selection results [17,18]. Clarke et al. [52] developed the maximum-likelihood population effects model with a covariate structure to explicitly model the correlated error structure in MRM, relying on restricted maximum likelihood (REML) to generate unbiased estimates of the variance components of the mixed effects models. However, Van Strien et al. [22] stated that model selection procedures based on AIC, AICc, and BIC should not be used to compare mixed models where parameter estimation is performed using REML with different fixed effects. Autocorrelated residuals resulting from various spatial processes (e.g., isolation-by-distance; IBD), can also severely affect regression results; potentially leading to spurious correlations in genetic analyses [53]. Recent studies demonstrated that including a vector of geographic distance to account for IBD does not sufficiently remove spatial autocorrelation [24,25]. Therefore, additional research is required to develop approaches that explicitly model the correlated error structure within vectors of pairwise distances *d*_*ij*_, as well as spatial autocorrelation among errors, and while still allowing the use of AIC, AICc, and BIC for model selection.

Depending on the nature of the data and question being addressed, a number of alternative analytical approaches are available that are not subject to the statistical issues associated with the analysis of pairwise distances [6]. Neighborhood-level approaches can be used to reduce pairwise distance matrices into node-level data vectors based on connectivity indices calculated for each focal site with all other sites within its local neighborhood; these represent either a single environmental factor or a resistance surface containing multiple factors [6]. Canonical redundancy analysis (RDA) [11], which has been shown to have greater power than Mantel-based approaches [25,45], can then be used to test for relationships between connectivity indices and measures of genetic diversity, genetic differentiation, a matrix of allele frequencies or a set of PCoA scores (distance based RDA) [54]. The functional connectivity (as measured by gene flow) through a network of observations can also be evaluated using gravity models which incorporate both at- and between-site variables, and allowing multiple parameters to be estimated from the sample data [55]. Alternatively, a predictor distance matrix may be used to define the error correlation structure in a node-based framework, using a table of allele frequencies rather than a genetic distance matrix as the response (e.g., [56]), either in a Bayesian context or with generalized linear mixed models (GLMM) [57,58]. There are, however, concerns about the validity of such covariance models [59], and it is unclear how valid model selection with multiple competing hypotheses could be performed in this type of analysis. While these approaches offer considerable promise for incorporating geographic and environmental distance into a single analysis, valid methods for statistical inference and model selection with landscape genetic data urgently require further development and evaluation.

## Conclusions

The development of statistically valid methods for comparing alternative hypotheses regarding the effects of landscape features on patterns of gene flow remains an important area of research in landscape genetics. Our results clearly demonstrated that AIC, AICc, and BIC were unable to reliably rank candidate models when applied with MRM, even under artificially ideal conditions, leading to systematically incorrect inferences. While e.g. AIC is known to overfit models in node-based analysis e.g. by including one predictor more than necessary, application to distance-based analysis typically resulted in AIC reversing the expected ranking of candidate models and preferentially selecting the full model with the maximum number of spurious predictors available. Methods for explicitly modeling the correlated error structure within vectors of pairwise distances *d*_*ij*_ resulting from non-independence of observations or spatial autocorrelation within a MRM framework are currently being explored, but additional research is needed to develop and test approaches that permit the use of AIC, AICc and/or BIC in a MRM framework [6]. Until these issues have been adequately addressed, we strongly discourage the continued used of AIC, AICc, and BIC with MRM.

## Supporting information

S1 File Simulation R code. The R code used to generate the node- and distance-based data vectors used in the simulation analysis.
(DOCX)

S2 File Evaluation of sample-size corrected model selection criteria. Description of a simple sample-size correction for AIC, AIC, and BIC (i.e., AICd, AICcd, and BICd) and its relative performance when used with MRM on distance matrices. Corrected measures were applied to the same data used in the manuscript. This is presented for illustration only and we do not recommend application of such correction in any form. Rather, we call on statisticians to help develop valid alternatives.
(DOCX)

S1 Fig Results from a single simulation run with sample-size corrected model selection criteria. (TIF)

S2 Fig Proportional selection of the correct model with sample-size corrected model selection criteria by means of MRM among 1000 simulated data sets with a different number of spurious predictors. (TIF)

S3 Fig Proportional selection of the correct model with sample-size corrected model selection criteria by means of MRM among 1000 simulated data sets for different levels of correlated predictors. (TIF)
