# Supplementary material for: Model selection with multiple regression on distance matrices leads to incorrect inferences
Source: PLoS One. 2017 Apr 13;12(4):e0175194. doi: 10.1371/journal.pone.0175194 (PMC5390996; doi:10.1371/journal.pone.0175194)
Supplement: S1 File — The R code used to generate the node- and distance-based data vectors used in the simulation analysis. (DOCX) [file pone.0175194.s001.docx]

###############################

# Testing AIC and corrections #

###############################

library(MASS)

library(MuMIn)

########################################################

# Function to simulate data and calculate all measures #

# for node- and distance-based analyses #

########################################################

sim.AIC<-function(N=100, multicol=0.0, Weights=c(0.60, 0.00, 0.00, 0.00, 0.00), Sort=FALSE)

{

# Define covariance matrix

std <- rep(1,6)

covmat <- matrix(multicol, 6, 6)

diag(covmat) <- 1

covmat[-1,1] <- covmat[1,-1] <- Weights

# Simulate predictors x1-x5 and response y, scale

df <- mvrnorm(N, mu = rep(0,6), Sigma = covmat)

df <- apply(df, 2, scale)

colnames(df) <- c("Y", paste("X", c(1:5), sep=""))

if(Sort==TRUE)

{

df <- df[,c(1:2, 2 + order(abs(cor(df))[-c(1:2),1], decreasing=TRUE))]

}

# Generate node-based data file for use in the regression model

Y<-df[,1]

X<-df[,2:6]

# Node-based analysis

AIC.values <- AICc.values <- BIC.values <- rep(NA, ncol(X))

for(i in 1:ncol(X))

{

modN <- lm(Y ~ X[,1:i])

AIC.values[i] <- AIC(modN)

AICc.values[i] <- AICc(modN)

BIC.values[i] <- BIC(modN)

}

# Convert to distance matrices

D.y <- as.vector(dist(df[,1]))

D.X <- matrix(NA, length(D.y), ncol(X))

for(k in 1:ncol(X)) {D.X[,k] <- as.vector(dist(X[,k]))}

Df <- cbind(D.y,D.X)

colnames(Df) <- c("Y", paste("X", c(1:5), sep=""))

# Distance-based analysis

AIC.values.dist <- AIC.calc.dist <- AIC.calc.corr <- AICc.values.dist <-AICc.calc.dist <- AICc.calc.corr<-

BIC.values.dist <-BIC.calc.dist <- BIC.calc.corr <- rep(NA, ncol(X))

for(i in 1:ncol(X))

{

modD <- lm(D.y ~ D.X[,1:i])

n <- nrow(D.X)

k <- i+2

p = k - 1

sigma <- sqrt(sum(residuals(modD)^2) / (n-p)) # Calculation of sigma from lm()

sigma.ML = sigma * sqrt((n - p)/n) # Convert to ML estimator (divided by n)

AIC.values.dist[i] <- AIC(modD)

AICc.values.dist[i] <- AICc(modD)

BIC.values.dist[i] <- BIC(modD)

# Recreate same values through calculation (for checking):

AIC.calc.dist[i] <- - 2 *(-(n/2) * log(2 * pi) -(n/2) * log(sigma.ML^2) -

1/(2 * sigma.ML^2)*sum(residuals(modD)^2)) + 2 * (i+2)

AICc.calc.dist[i] <- - 2 *(-(n/2) * log(2 * pi) -(n/2) * log(sigma.ML^2) -

1/(2 * sigma.ML^2)*sum(residuals(modD)^2)) + (2 * (i+2)) + 2*(i+2)*((i+2)+1)/(n-(i+2)-1)

BIC.calc.dist[i] <- - 2 *(-(n/2) * log(2 * pi) -(n/2) * log(sigma.ML^2) -

1/(2 * sigma.ML^2)*sum(residuals(modD)^2)) + (i+2) * log(n)

# Distance-corrected measures (not recommended for use!)

AIC.calc.corr[i] <- - 2 *(-(N/2) * log(2 * pi) -(N/2) * log(sigma.ML^2) -

1/(2 * sigma.ML^2)*sum(residuals(modD)^2)) + 2 * (i+2)

AICc.calc.corr[i] <- - 2 *(-(N/2) * log(2 * pi) -(N/2) * log(sigma.ML^2) -

1/(2 * sigma.ML^2)*sum(residuals(modD)^2)) + (2 * (i+2)) + 2*(i+2)*((i+2)+1)/(N-(i+2)-1)

BIC.calc.corr[i] <- - 2 *(-(N/2) * log(2 * pi) -(N/2) * log(sigma.ML^2) -

1/(2 * sigma.ML^2)*sum(residuals(modD)^2)) + (i+2) * log(N)

}

res <- data.frame(AIC.values, AIC.values.dist, AIC.calc.corr,

AICc.values, AICc.values.dist, AICc.calc.corr,

BIC.values, BIC.values.dist, BIC.calc.corr)

#list(Table=res, Cor= c(Node1=round(cor(df)[1,2],2), Dist=round(as.vector(cor(D.y, D.X[,1])),2)),nodedata=df,distdata=Df)

list(Table=res, Cor.node= cor(Y, X), Cor.dist=cor(D.y, D.X), nodedata=df,distdata=Df)

}

########################################################

# Illustrate single simulation results #

########################################################

############################################################################### Single simulation run

##############################################################################

#set.seed(40) # Use this to recreate example from Fig. 1

test06 <- sim.AIC(N=100, multicol=0.0, Weights=c(0.60, 0.00, 0.00, 0.00, 0.00), Sort=TRUE) # Low correlation set

test08 <- sim.AIC(N=100, multicol=0.0, Weights=c(0.80, 0.00, 0.00, 0.00, 0.00), Sort=TRUE) # High correlation set

##############################################################################

# Generate data table for absolute values, deltas and model weights

##############################################################################

test06$Table

Delta06 <- t(t(test06$Table) - apply(test06$Table, 2, min))

Weights06 <- t(t(exp(-0.5 *Delta06)) / apply(exp(-0.5 *Delta06), 2, sum))

test08$Table

Delta08 <- t(t(test08$Table) - apply(test08$Table, 2, min))

Weights08 <- t(t(exp(-0.5 *Delta08)) / apply(exp(-0.5 *Delta08), 2, sum))

##############################################################################

# Figure 1 AIC example: absolute vals, deltas, or model weights for node, distance low corr and distance high corr

##############################################################################

op <- par(mfrow = c(3,3), oma = c(9,6,6,4) + 0.2, mar = c(0,2.0,1.5,4.0) + 0.2)

plot(c(0:4), test06$Table[,"AIC.values"], type="b", col=1, main="", xlab="", ylab="",

ylim=c(min(test06$Table[,"AIC.values"]),max(test06$Table[,"AIC.values"])), cex.axis=1.25, xaxt="n")

axis(side=1, at=0:4, labels=FALSE)

mtext("Node", 3, 1.0, cex=1.0, outer=FALSE)

mtext("Values", side = 2, line = 3, cex = 1.0, col = NULL)

plot(c(0:4), test06$Table[,"AIC.values.dist"], type="b", col=1, main="", xlab="", ylab="",

ylim=c(min(test06$Table[,"AIC.values.dist"]),max(test06$Table[,"AIC.values.dist"])), cex.axis=1.25, xaxt="n")

axis(side=1, at=0:4, labels=FALSE)

mtext("Dist (LC)", 3, 1.0, cex=1.0, outer=FALSE)

plot(c(0:4), test08$Table[,"AIC.values.dist"], type="b", col=1, main="", xlab="", ylab="",

ylim=c(min(test08$Table[,"AIC.values.dist"]),max(test08$Table[,"AIC.values.dist"])), cex.axis=1.25, xaxt="n")

axis(side=1, at=0:4, cex.axis=1.25, labels=FALSE)

mtext("Dist (HC)", 3, 1.0, cex=1.0, outer=FALSE)

plot(c(0:4), Delta06[,"AIC.values"], type="b", col=1, main="", xlab="", ylab="",

ylim=c(min(Delta06[,"AIC.values"]),max(Delta06[,"AIC.values"])), cex.axis=1.25, xaxt="n")

axis(side=1, at=0:4, labels=FALSE)

mtext("Deltas", side = 2, line = 3, cex = 1.0, col = NULL)

plot(c(0:4), Delta06[,"AIC.values.dist"], type="b", col=1, main="", xlab="",

ylab="", ylim=c(min(Delta06[,"AIC.values.dist"]),max(Delta06[,"AIC.values.dist"])), cex.axis=1.25, xaxt="n")

axis(side=1, at=0:4, labels=FALSE)

plot(c(0:4), Delta08[,"AIC.values.dist"], type="b", col=1, main="", xlab="", ylab="",

ylim=c(min(Delta08[,"AIC.values.dist"]),max(Delta08[,"AIC.values.dist"])), cex.axis=1.25, xaxt="n")

axis(side=1, at=0:4, cex.axis=1.25, labels=FALSE)

plot(c(0:4), Weights06[,"AIC.values"], type="b", col=1, main="", xlab="", ylab="",

ylim=c(0,1), cex.axis=1.25, xaxt="n")

axis(side=1, at=0:4, cex.axis=1.25, labels=TRUE)

mtext("Weights", side = 2, line = 3, cex = 1.0, col = NULL)

plot(c(0:4), Weights06[,"AIC.values.dist"], type="b", col=1, main="", xlab="", ylab="",

ylim=c(0,1), cex.axis=1.25, xaxt="n")

axis(side=1, at=0:4, cex.axis=1.25, labels=TRUE)

mtext("Number of spurious variables added", side = 1, line = 3, adj = 0.5, cex = 1.0, col = NULL)

plot(c(0:4), Weights08[,"AIC.values.dist"], type="b", col=1, main="", xlab="", ylab="",

ylim=c(0,1), cex.axis=1.25, xaxt="n")

axis(side=1, at=0:4, cex.axis=1.25, labels=TRUE)

par(op)

####################################################################

# Multiple runs: Compare 0.6 to 0.8 with change in sample size n #

####################################################################

############################################################################### Run simulation for correlation 0.6 and 0.8

##############################################################################

R = 1000

Results1.d1 <- Results1.d2 <-Results1.d3 <-Results1.d4 <-Results1.d5 <-Results1.w <- matrix(NA, R, 9,

dimnames=list(NULL, c("AIC.node","AIC.dist","AIC.corr","AICc.node","AICc.dist","AICc.corr",

"BIC.node","BIC.dist","BIC.corr")))

Results1.cor <- matrix(NA, R, 2, dimnames=list(NULL, c("Node","Dist")))

for(r in 1:R)

{

test1 <- sim.AIC(N=300, multicol=0.0, Weights=c(0.60, 0.00, 0.00, 0.00, 0.00), Sort=TRUE)

Delta1 <- t(t(test1$Table) - apply(test1$Table, 2, min))

Results1.d1[r,] <- as.numeric(Delta1[1,] == 0) # Correct model ranked best

Results1.d2[r,] <- as.numeric(Delta1[2,] == 0)

Results1.d3[r,] <- as.numeric(Delta1[3,] == 0)

Results1.d4[r,] <- as.numeric(Delta1[4,] == 0)

Results1.d5[r,] <- as.numeric(Delta1[5,] == 0)

Weights1 <- t(t(exp(-0.5 *Delta1)) / apply(exp(-0.5 *Delta1), 2, sum))

Results1.w[r,] <- Weights1[1,] # Weight of correct model

Results1.cor[r,] <- c(test1$Cor.node[1], test1$Cor.dist[1])

cat(r)

}

Results2.d1 <- Results2.d2 <-Results2.d3 <-Results2.d4 <-Results2.d5 <-Results2.w <- matrix(NA, R, 9,

dimnames=list(NULL, c("AIC.node","AIC.dist","AIC.corr","AICc.node","AICc.dist","AICc.corr",

"BIC.node","BIC.dist","BIC.corr")))

Results2.cor <- matrix(NA, R, 2, dimnames=list(NULL, c("Node","Dist")))

for(r in 1:R)

{

test2 <- sim.AIC(N=300, multicol=0.0, Weights=c(0.80, 0.00, 0.00, 0.00, 0.00), Sort=TRUE)

Delta2 <- t(t(test2$Table) - apply(test2$Table, 2, min))

Results2.d1[r,] <- as.numeric(Delta2[1,] == 0)

Results2.d2[r,] <- as.numeric(Delta2[2,] == 0)

Results2.d3[r,] <- as.numeric(Delta2[3,] == 0)

Results2.d4[r,] <- as.numeric(Delta2[4,] == 0)

Results2.d5[r,] <- as.numeric(Delta2[5,] == 0)

Weights2 <- t(t(exp(-0.5 *Delta2)) / apply(exp(-0.5 *Delta2), 2, sum))

Results2.w[r,] <- Weights2[1,] # Weight of correct model

Results2.cor[r,] <- c(test2$Cor.node[1], test2$Cor.dist[1])

cat(r)

}

##############################################################################

# Check whether cor.dist with 0.8 is comparable to cor. node with 0.6

##############################################################################

# Only run this with n=100

op <- par(mfrow = c(1,1), oma = c(9,6,6,4) + 0.2, mar = c(0,2.0,1.5,4.0) + 0.2)

boxplot(cbind(Results1.cor, Results2.cor), las=1, lty = 1,

names=c("Node (LC)","Dist (LC)","Node (HC)","Dist (HC)"))

lines(c(0,10), c(0.60, 0.60), lty = 2)

mtext("Empirical Correaltion", side = 2, line = 3.0, cex=1.0, outer=FALSE)

mtext("Figure 1.", at = c(0,0), side = 1, line = 2.5, cex=1.0, outer=TRUE)

# Extract mean and sd of correlations:

round(rbind(Mean=apply(cbind(Results1.cor, Results2.cor[,-1]), 2, mean),

SD=apply(cbind(Results1.cor, Results2.cor[,-1]), 2, sd)),3)

##############################################################################

# Simulation output data for spurious variable analysis:

# node, distance low correlation, and distance high correlation for n=30, 100, 300

##############################################################################

# First run N = 30

Delta0.6N30<-rbind(colMeans(Results1.d1),colMeans(Results1.d2),colMeans(Results1.d3),

colMeans(Results1.d4),colMeans(Results1.d5))

Delta0.8N30<-rbind(colMeans(Results2.d1),colMeans(Results2.d2),colMeans(Results2.d3),

colMeans(Results2.d4),colMeans(Results2.d5))

AICDeltaN30<-cbind(Delta0.8N30[,2],Delta0.6N30[,2:1])

AICcDeltaN30<-cbind(Delta0.8N30[,5],Delta0.6N30[,5:4])

BICDeltaN30<-cbind(Delta0.8N30[,8],Delta0.6N30[,8:7])

# Second run N = 100

Delta0.6N100<-rbind(colMeans(Results1.d1),colMeans(Results1.d2),colMeans(Results1.d3),

colMeans(Results1.d4),colMeans(Results1.d5))

Delta0.8N100<-rbind(colMeans(Results2.d1),colMeans(Results2.d2),colMeans(Results2.d3),

colMeans(Results2.d4),colMeans(Results2.d5))

AICDeltaN100<-cbind(Delta0.8N100[,2],Delta0.6N100[,2:1])

AICcDeltaN100<-cbind(Delta0.8N100[,5],Delta0.6N100[,5:4])

BICDeltaN100<-cbind(Delta0.8N100[,8],Delta0.6N100[,8:7])

# Third run N = 300

Delta0.6N300<-rbind(colMeans(Results1.d1),colMeans(Results1.d2),colMeans(Results1.d3),

colMeans(Results1.d4),colMeans(Results1.d5))

Delta0.8N300<-rbind(colMeans(Results2.d1),colMeans(Results2.d2),colMeans(Results2.d3),

colMeans(Results2.d4),colMeans(Results2.d5))

AICDeltaN300<-cbind(Delta0.8N300[,2],Delta0.6N300[,2:1])

AICcDeltaN300<-cbind(Delta0.8N300[,5],Delta0.6N300[,5:4])

BICDeltaN300<-cbind(Delta0.8N300[,8],Delta0.6N300[,8:7])

##############################################################################

# Figure 2 spurious variable analysis: node, distance low correlation,

# and distance high correlation for n=30,100,300

##############################################################################

op <- par(mfcol = c(3,3), oma = c(9,8,6,6) + 0.2, mar = c(0,1.5,1.5,0.5) + 0.2)

barplot(AICDeltaN30,main="n = 30",names.arg=c("Dist (HC)","Dist (LC)","Node (LC)"),horiz=TRUE,

xlab="", ylab="",las=1 ,cex.axis=3.0, cex=1.2,xaxt="n")

axis(side=1, at=c(0.0,0.2,0.4,0.6,0.8,1.0), labels=FALSE)

barplot(AICcDeltaN30,main=NULL,names.arg=c("Dist (HC)","Dist (LC)","Node (LC)"),horiz=TRUE,

xlab="", ylab="",las=1,cex.axis=3.0, cex=1.2, xaxt="n")

axis(side=1, at=c(0.0,0.2,0.4,0.6,0.8,1.0), labels=FALSE)

barplot(BICDeltaN30,main=NULL,names.arg=c("Dist (HC)","Dist (LC)","Node (LC)"),horiz=TRUE,

las=1,cex.axis=1.5, cex=1.2)

barplot(AICDeltaN100,main="n = 100",horiz=TRUE,xlab="",cex.axis=1.25,xaxt="n", yaxt="n")

axis(side=1, at=c(0.0,0.2,0.4,0.6,0.8,1.0), labels=FALSE)

barplot(AICcDeltaN100,main=NULL,horiz=TRUE, xlab="", cex.axis=5, xaxt="n", yaxt="n")

axis(side=1, at=c(0.0,0.2,0.4,0.6,0.8,1.0), labels=FALSE)

barplot(BICDeltaN100,main=NULL,horiz=TRUE,yaxt="n", ,cex.axis=1.5)

mtext("Proportion of Simulations", side = 1, line = 3, adj = 0.5, cex = 1.0, col = NULL)

barplot(AICDeltaN300,main="n = 300",horiz=TRUE, xlab="",cex.axis=1.25,xaxt="n", yaxt="n")

axis(side=1, at=c(0.0,0.2,0.4,0.6,0.8,1.0), labels=FALSE)

mtext("AIC", 4, 1.0, cex=1.0, las=1,outer=FALSE)

barplot(AICcDeltaN300,main=NULL,horiz=TRUE,xlab="", cex.axis=5, xaxt="n", yaxt="n")

axis(side=1, at=c(0.0,0.2,0.4,0.6,0.8,1.0), labels=FALSE)

mtext("AICc", 4, 1.0, cex=1.0, las=1,outer=FALSE)

barplot(BICDeltaN300,main=NULL,horiz=TRUE,yaxt="n",cex.axis=1.5)

mtext("BIC", 4, 1.0, cex=1.0, las=1,outer=FALSE)

par(op)

########################################################

# END #

########################################################
