# Supplementary material for: Model selection with multiple regression on distance matrices leads to incorrect inferences
Source: PLoS One. 2017 Apr 13;12(4):e0175194. doi: 10.1371/journal.pone.0175194 (PMC5390996; doi:10.1371/journal.pone.0175194)
Supplement: S2 File — Description of a simple sample-size correction for AIC, AIC, and BIC (i.e., AICd, AICcd, and BICd) and its relative performance when used with MRM on distance matrices. Corrected measures were applied to the same data used in the manuscript. This is presented for illustration only and we do not recommend application of such correction in any form. Rather, we call on statisticians to help develop valid alternatives. (DOCX) [file pone.0175194.s002.docx]

**S2 File: Evaluation of sample-size corrected model selection criteria**

**Model selection criteria calculations**

AIC, AICc, and BIC values can be calculated using ordinary least squares (OLS) estimation, assuming model residuals are independent, normally distributed with constant variance; or more generally, using maximum likelihood estimation procedures with the following formulas [1,2]:

$AIC= -2\log\left( L\left( \hat{\theta} | data, g_{i} \right) \right)+2K$ (1) ${AIC}_{c}= -2\log\left( L\left( \hat{\theta} | data, g_{i} \right) \right)+2K+\frac{2K\left( K-1 \right)}{\left( n-k-1 \right)}$ (2)

$BIC= -2\log\left( L\left( \hat{\theta} | data, g_{i} \right) \right)+K\log\left( n \right)$ (3)

where $-2log \left( L\left( \hat{\theta} | data, g_{i} \right) \right)$ is the numerical value of the log-likelihood at its maximum point for a model *g_i_* given the data, and thus, provides a measure of model fit. The latter portion of the formula represents an asymptotic bias-correction term (i.e., penalty) designed to account for the change in model fit related to the numbers of parameters *K* estimated from the data (*K* = *p* + 2; number of predictors *p* plus two to account for the intercept and the error term) for a given sample size *n*. The log-likelihood for a linear regression model is calculated as:

$-2log \left( L\left( \hat{\theta} | data, g_{i} \right) \right) =-\frac{n}{2}\log\left( 2\pi\right)-\frac{n}{2}\log\left( \hat{\sigma}^{2} \right)-\frac{1}{\left( 2\hat{\sigma}^{2} \right)}\sum_{i=1}^{n} \epsilon_{i}^{2}$ (4)

where $\hat{\sigma}^{2}$is the maximum likelihood estimator of model variance (RSS/n) and $\sum_{i=1}^{n} \epsilon_{i}^{2}$ is the residual sum of squares (RSS) from the linear model fit to the original data with *n* observations.

**Distance-corrected measures AIC, AICcd, and BICd**

In MRM, the *n* original observations are represented by vector of *N* = *n* (*n* – 1) / 2 non-redundant pairwise differences. For instance, ignoring any violations of assumptions regarding error distributions, equation 3 would thus be calculated as:

${BIC}_{d}= -2\left( -\frac{n}{2}\log\left( 2\pi\right)-\frac{n}{2}\log\left( \hat{\sigma}_{d}^{2} \right)-\frac{1}{\left( 2\hat{\sigma}_{d}^{2} \right)}\sum_{i=1}^{N} \epsilon_{i}^{2} \right)+K\log\left( n \right)$

where $\hat{\sigma}_{d}^{2}$ = RSS_d_  / *N* is the maximum likelihood estimator of model variance of the MRM model (i.e., of the distance values) and $\sum_{i=1}^{N} \epsilon_{i}^{2}$ is the residual sum of squares (RSS_d_) from the linear model fitted to the distance transformed data with *N* observations.

Here we evaluate the effect of a simple sample size correction, where distance-corrected measures AICd, AICcd and BICd are calculated by replacing *N* by *n* as follows:

${AIC}_{d}= -2\left( -\frac{n}{2}\log\left( 2\pi\right)-\frac{n}{2}\log\left( \hat{\sigma}_{d}^{2} \right)-\frac{1}{\left( 2\hat{\sigma}_{d}^{2} \right)}\sum_{i=1}^{N} \epsilon_{i}^{2} \right)+2K$ (5)

${AIC}_{cd}= -2\left( -\frac{n}{2}\log\left( 2\pi\right)-\frac{n}{2}\log\left( \hat{\sigma}_{d}^{2} \right)-\frac{1}{\left( 2\hat{\sigma}_{d}^{2} \right)}\sum_{i=1}^{N} \epsilon_{i}^{2} \right)+2K+\frac{2K(K-1)}{(n-k-1)}$ (6)

${BIC}_{d}= -2\left( -\frac{n}{2}\log\left( 2\pi\right)-\frac{n}{2}\log\left( \hat{\sigma}_{d}^{2} \right)-\frac{1}{\left( 2\hat{\sigma}_{d}^{2} \right)}\sum_{i=1}^{N} \epsilon_{i}^{2} \right)+K\log\left( n \right)$ (7)

where $\hat{\sigma}_{d}^{2}$ = RSS_d_  / *N* is retained, as RSS_d_ is fundamentally different from the RSS obtained from regression of the original *n* observations [3], and replacing *N* with *n* here would lead to a meaningless quantity. The proposed corrections were developed to specifically account for the effect of inflated sample size, but do not make any attempt to correct for correlated error structure [4,5], which is beyond the scope of this analysis.

**Performance with simulated data**

We repeated the analyses of the main paper with these distance-corrected model selection criteria. Results from the same single simulation run as shown in the original S1_Fig indicated that the distance-corrected measures, AICd, AICcd, and BICd, ranked models correctly and similarly to the node-based regression analysis (S1_Fig). Delta values Δ_i_ were on a similar scale and so were the model weights *w_i_*; which decreased as expected with each additional variable added to the model, for both levels of correlation between *x*_1_ and *y*.

**S1_Fig**: **Results from a single simulation run with sample-size corrected model selection criteria.** Results from a single simulation run showing the absolute values (top row), delta values Δ_i_ (middle row), and model weights *w_i_* (bottom row) for AICd (left column), AICcd (middle column), and BICd (right column) as a function of the number of spurious predictors added sequentially to the correct model with a single meaningful predictor *x*_1_. Circles indicate results from the low correlation data set, triangles from the high correlation data set.

Across the 1000 replicate simulations with four spurious predictors added to the correct single-predictor model, the distance-corrected measures AICd, AICcd, and BICd selected the correct model in more than 98 percent of simulations regardless of sample size *n* or strength of correlation (S2_Fig).

**S2_Fig**: **Proportional selection of the correct model with sample-size corrected model selection criteria by means of MRM among 1000 simulated data sets with a different number of spurious predictors.** The proportion of 1000 simulated data sets where each of the five candidate models was selected as the best model using AIC (top row), AICc (middle row), and BIC (bottom row) with three different sample sizes if *n* = 30 (left column), 100 (middle column), 300 (right column) for the node-based analysis with low correlation (Node LC), the distance-based analysis with low correlation (Dist LC), the distance-corrected analysis with low correlation (Corr LC), ), the distance-based analysis with high correlation (Dist HC), and the distance-corrected analysis with high correlation (Corr HC). The correct (i.e., data generating) model included only the single meaningful predictor $x_{1}$ (black), whereas, the four additional models contained the single meaningful predictor $x_{1}$ and one (dark grey), two (medium dark grey), three (medium light grey), and four (light grey) spurious variables ($x_{2}- x_{5}$).

In the second set of simulations with three meaningful predictors with tapered effects (i.e., decreasing strength of correlation with *y*), the performance of the distance-corrected measures AICd, AICcd, and BICd varied considerably depending on sample size *n* and strength of correlation (S3_Fig). For the low correlation data, AICd, AICcd, and BICd failed to identify the correct model regardless of sample size *n*; selecting instead the single variable model in 72 to 98 percent of simulations. For the high correlation (ρ_xy_ = 0.8) data, AICd, AICcd, and BICd performed similarly to the node-based analysis for small sample size *n* = 30, often selecting too few predictors, and even outperformed the node-based regression analysis with large sample size of *n* = 300.

**S3_Fig**: **Proportional selection of the correct model with sample-size corrected model selection criteria by means of MRM among 1000 simulated data sets for different levels of correlated predictors.** The proportion of 1000 simulated data sets where each of the five candidate models was selected as the best model using AIC (top row), AICc (middle row), and BIC (bottom row) with three different sample sizes of *n* = 30 (left column), 100 (middle column), 300 (right column) for the node-based analysis with low correlation (Node LC), the distance-based analysis with low correlation (Dist LC), the distance-corrected analysis with low correlation (Corr LC), the distance-based analysis with high correlation (Dist HC), and the distance-corrected analysis with high correlation (Corr HC). We were primarily interested in determining whether AIC, AICc, and BIC selected the correct model containing three meaningful variables with tapering effects (black) or selected an underfitted (dark grey) or overfitted (light grey) model.

These results suggest that a sample size correction may substantially improve performance of AIC, AICc and BIC when applied to MRM but cannot be recommended as it does not adequately address the problem of unknown degrees of freedom [6]. Moreover, our simulations reflect an artificially simple situation that is suitable for demonstrating the problems of uncorrected model selection criteria, but lacks much of the complexity of real data, and it is unclear under what conditions a sample size correction is likely to produce reliable results.

**References**

1. Burnham KP, Anderson DR. Model selection and multimodel inference: A practical information-theoretic approach. Heidelberg and New York: Springer; 2002.
2. Burnham KP, Anderson DR. Multimodel inference: Understanding AIC and BIC in model selection. Sociol Methods Res. 2004; 33: 261-304.
3. Legendre P, Fortin MJ. Comparison of the Mantel test and alternative approaches for detecting complex multivariate relationships in the spatial analysis of genetic data. Mol Ecol Res. 2010; 10: 831-844.
4. Clarke RT, Rothery P, Raybould AF. Confidence limits for regression relationships between distance matrices: Estimating gene flow with distance. J Agric Biol Environ Stat. 2002; 7: 361-372.
5. Van Strien MJ, Keller D, Holderegger R. A new analytical approach to landscape genetic modelling: Least-cost transect analysis and linear mixed models. Mol Ecol. 2012; 21: 4010-4023.
6. Legendre P, Fortin MJ. Spatial pattern and ecological analysis. Vegetatio. 1989; 80:107-138.
