# Supplementary figures and images for: Model selection with multiple regression on distance matrices leads to incorrect inferences
Source: PLoS One. 2017 Apr 13;12(4):e0175194. doi: 10.1371/journal.pone.0175194 (PMC5390996; doi:10.1371/journal.pone.0175194)

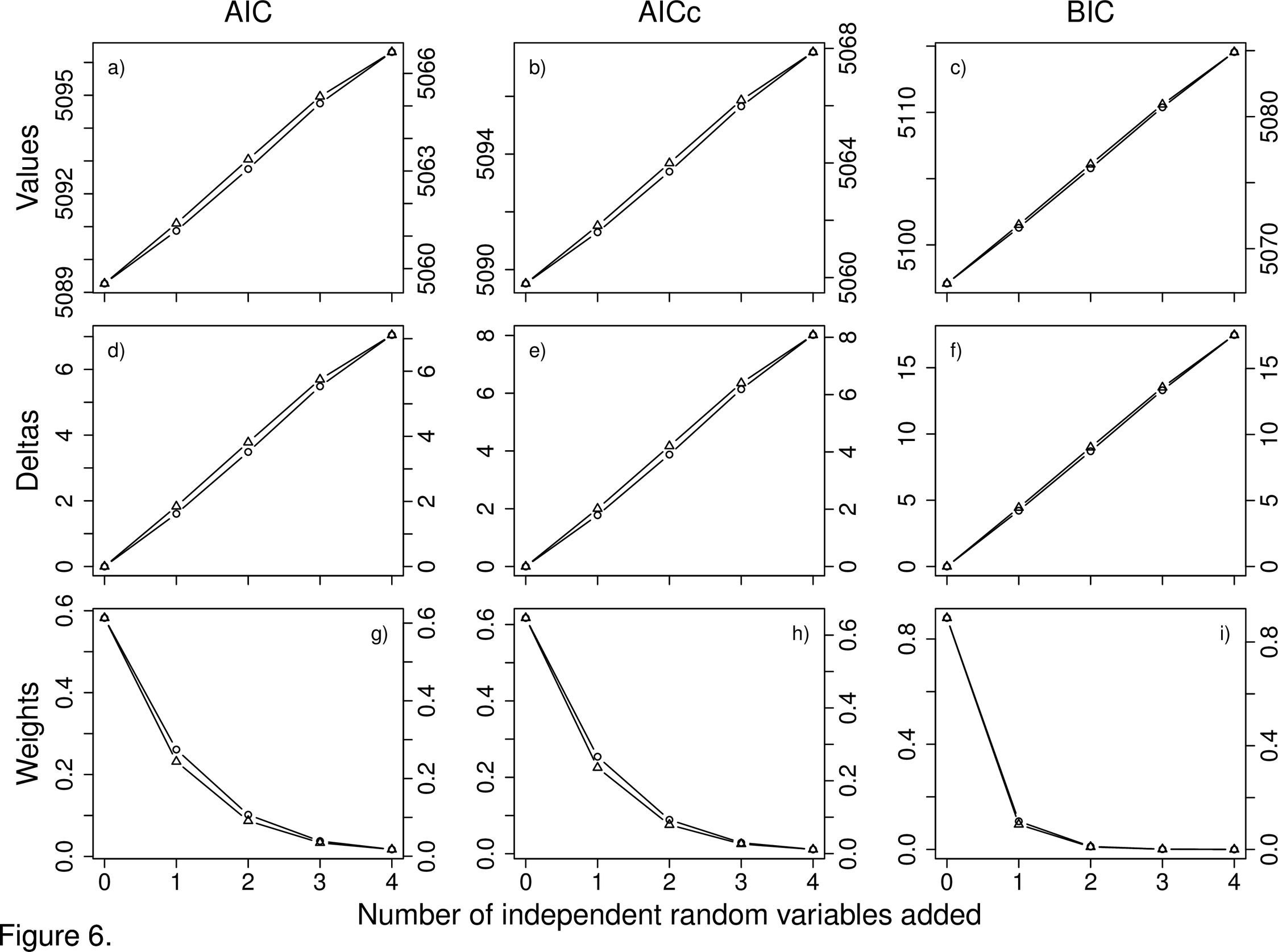

Supplement: S1 Fig — (TIF) [file pone.0175194.s003.tif]

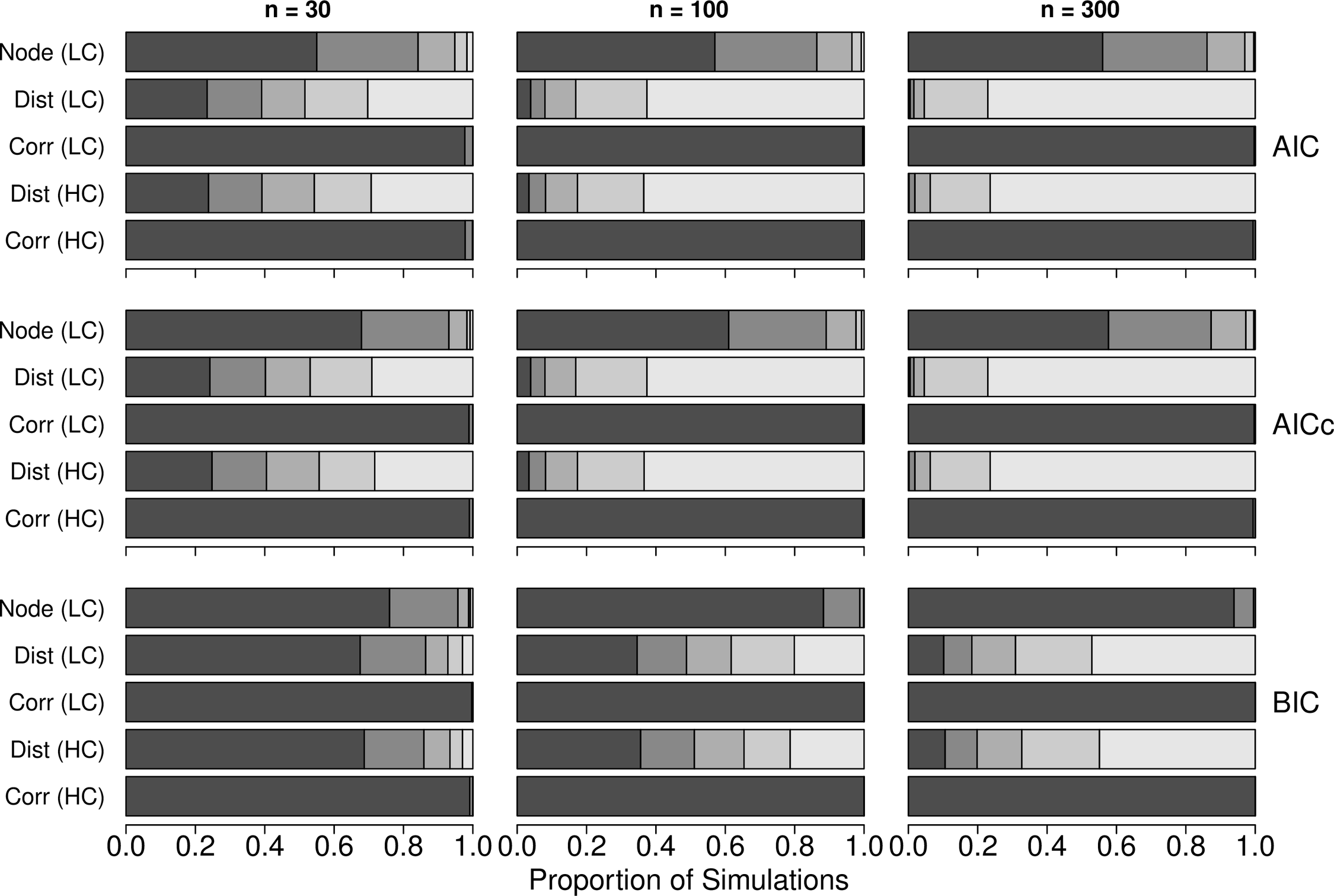

Supplement: S2 Fig — (TIF) [file pone.0175194.s004.tif]

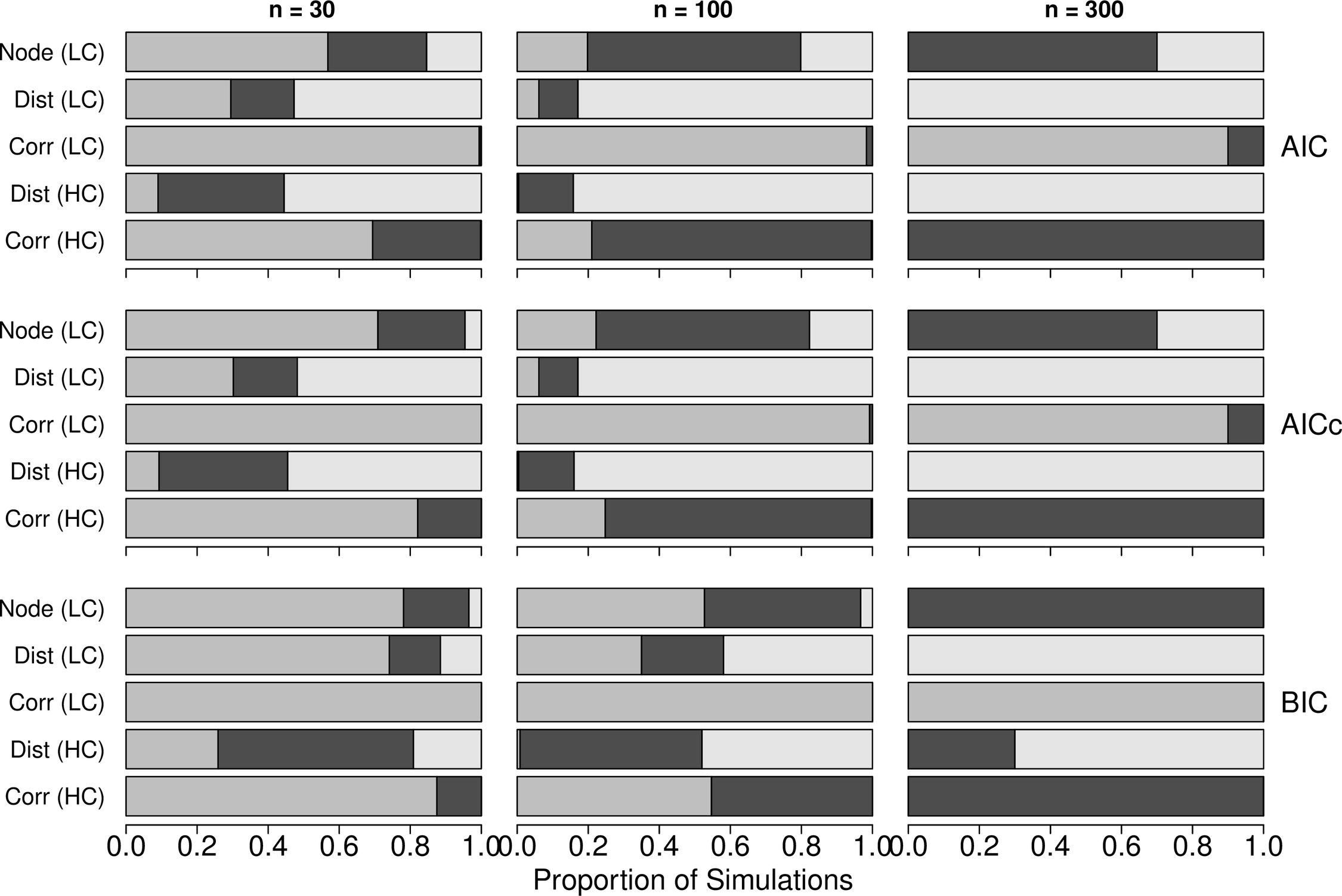

Supplement: S3 Fig — (TIF) [file pone.0175194.s005.tif]
